# Effect of *Glomus intraradices* on root morphology, biomass production and phosphorous use efficiency of Chinese fir seedlings under low phosphorus stress

**DOI:** 10.3389/fpls.2022.1095772

**Published:** 2023-01-06

**Authors:** Yunlong Tian, Jingjing Xu, Xiaoqian Lian, Bo Wei, Xiangqing Ma, Pengfei Wu

**Affiliations:** College of Forestry, Fujian Agriculture and Forestry University, Fuzhou, China

**Keywords:** Chinese fir, arbuscular mycorrhizal fungi, root morphology, abiotic stress, phosphorus utilization strategy

## Abstract

**Introduction:**

Available phosphorus (P) scarcity in the highly weathered soils of the subtropical forests in southern China is a serious concern. To ensure whether inoculation of arbuscular mycorrhizal fungi (AMF) with Chinese fir (*Cunninghamia lanceolata*) under low P stress conditions could promote its growth and P utilization capacity, an indoor pot simulation experiment was carried out with the different P supply treatments and Chinese fir seedlings as the tested material.

**Methods:**

The experiment had two P supply treatments, no P supply (P0, 0 mmol·L^-1^ KH_2_PO_4_) and normal P supply (P1, 1.0 mmol·L^-1^ KH_2_PO_4_). The seedling in each P supply treatment was inoculated with *Glomus intraradices* (Gi), a widespread species of AMF in the natural environment, and with no AMF inoculation as a control treatment (CK). The Gi infection rate in the root system, root cortex tissue dissolution rate, root morphological indexes and biomass, whole plant P use efficiency, and root P use efficiency of Chinese fir were determined under different treatment conditions.

**Results and Discussion:**

The results showed that P0 treatment significantly increased the Gi infection rate (*p*< 0.05). After inoculating AMF with different P supply treatments, the root cortex tissue dissolution rate was considerably enhanced. In contrast, the Chinese fir’s root length and surface area were reduced; however, the root volume did not change significantly. The average root diameter in the P0 treatment and inoculated with AMF was significantly more prominent than in the uninoculated treatment (*p<* 0.05). The root biomass and root-to-shoot ratio at different P supply treatments were significantly higher in the Gi infection treatment than in the CK group. Under different P supply treatments, root inoculation with Gi promoted root P use efficiency and whole plant P use efficiency. In conclusion, low P stress condition promoted the colonization of AMF in the root system, increased the dissolution of root cortex tissue, root volume, and the average diameter, and promoted root biomass accumulation and P use efficiency.

## 1 Introduction

Arbuscular mycorrhizal fungi (AMF) are widely distributed in the natural environment, forming a mutually beneficial symbiosis through soil infestation of most terrestrial plant roots (Goltapeh et al., 2008). Numerous studies have shown that AMF colonization facilitates phosphorus (P) uptake for the plant roots (Smith et al., 2004; Rooney et al., 2011). In return, plants transfer fixed carbon (C), sugars, and lipids to satisfy the symbiotic fungi growth (Ferrol et al., 2019). Liu et al. (2014) found that inoculation with AMF promoted Canada poplar’s (*Populus × canadensis*) growth and physiological activity. However, the positive effect of symbiotic fungi on the hosts varied to different soil conditions, e.g., the nitrogen (N) forms in the culture substrate can remarkably adjust the interaction efficiency between forest trees and fungi (Qin et al., 2017). As one of the leading nutrient elements for tree growth, P is involved in tree growth and development in various ways and plays a vital role in plantation production (Wu et al., 2020). It is crucial to carry out studies to uncover the effectiveness of AMF on the P use efficiency of trees. Previous studies showed that low available P in the highly weathered soil strongly limits the tree’s average growth, especially to changes in natural conditions, including the soil’s physical and chemical properties, climate, and topography. Moreover, inappropriate anthropogenic nurturing practices have exacerbated the P heterogeneous distribution in the soil, resulting in more difficulty for the root system to forage the P nutrient efficiently (Zhang et al., 2013; Zou et al., 2015; Yang, 2018). The biased investment in roots by forest trees inevitably leads to nutrient depletion, limiting tree growth and development (Mou et al., 2013). Therefore, it has become an urgent problem to maintain the high productivity of plantations in the long term in a practical way, combing with the contribution of AMF.

Chinese fir (*Cunninghamia lanceolata*) is an important plantation species widely used in southern China (Farooq et al., 2019). Its continuous planting further leads to decreased soil available P content, which leads to reduced forest productivity. It was found that under low P stress, some Chinese fir genotypes or clones could improve P foraging ability through root elongation and radial proliferation or through differentiation of fine roots, root secretion, and root cortical aerenchyma (RCA) (Wu et al., 2017; Yu et al., 2017; Wu et al., 2018; Zou et al., 2018). Studies on the symbiotic relationship between Chinese fir and AMF also showed that the species of the *Glomus* was susceptible to symbiosis with Chinese fir root systems (Li et al., 2019; Lu et al., 2019; Guo et al., 2022), and AMF colonization can promote the growth of Chinese fir (Li et al., 2011). So, does the symbiotic relationship between Chinese fir and AMF help Chinese fir to resist low P stress? Is there a significant difference in the symbiotic relationship between Chinese fir and AMF under different P supply treatments? We hypothesize that: (1) under low P stress, Chinese fir enhances the relationship with AMF, and (2) colonization of AMF in Chinese fir roots is helpful to improve P use efficiency, maintain normal growth and resist low P stress. The improvement of P use efficiency may be related to the morphological changes of roots and the formation of RCA by cortex tissue dissolution.

The present study chose 1.5-year Chinese fir seedlings (clone No. 41) as the test material. We set up two P supply treatments, no P supply 0 mmol·L^-1^KH_2_PO_4_ (0 ppm) and normal P supply 1.0 mmol·L^-1^KH_2_PO_4_ (136.09 ppm) in an indoor experiment. The seedlings in each P supply treatment were inoculated with *Glomus intraradices* (Gi) and no AMF inoculation (CK). We measured the infection rate of Gi in root systems, root morphological indexes, plant biomass production and allocation, P use efficiency, and root cortex tissue dissolution rate. This study will provide a theoretical basis for the in-depth analysis of soil phosphorus utilization by Chinese fir under symbiotic conditions with AMF mycorrhizae.

## 2 Materials and methods

### 2.1 Plant materials and experimental design

1.5-year-old Chinese fir seedlings (Clone No.41) taken from the Chinese Fir Engineering Technology Research Center of the State Forestry and Grassland Administration were selected as the test material. The seedlings’ average height and root collar diameter were 19.1 ± 0.5 cm and 3.03 ± 0.08 mm, respectively. Seedlings were cultivated in a nursery for 17 months, then transplanted and stored in a sand-bed for 1 month, supplying appropriate watering according to weather conditions at the College of Forestry, Fujian Agriculture and Forestry University (Li et al., 2022). Before AMF inoculation treatment, three Chinese fir seedlings were randomly taken from the experimental materials, and their roots were examined under the microscope. It was determined that there was no AMF colonization in the roots (**Figure S1**).

A polyethylene pot of 20 cm in length, 16 cm in width, and 20 cm in height were set up in a greenhouse at Fujian Agriculture and Forestry University. The pots were soaked in 0.1 g·L^-1^ KMnO_4_ solution for 20 min before use, rinsed with clean water, dried, and set aside. One Chinese fir seedling was planted in each pot after being thoroughly disinfected with 0.5% NaClO solution on the root surface for 5s, then repeatedly rinsed with sterile water to reduce exogenous fungal infection.

According to the nutrient characteristics of subtropical red soil in southern China (Chen, 2003), a normal P supply treatment (1.0 mmol·L^-1^KH_2_PO_4_ (136.09 ppm), P1) was set up to ensure that Chinese fir seedlings in this treatment group to grow under rich P environment (Wu et al., 2011; 2018). After P1 addition, the available P content in the mixed matrix was 4.51 ± 0.02 mg·kg^−1^ (1 mg·kg^−1^ = 1 ppm). The second treatment was no P supply treatment ((0 mmol·L^-1^KH_2_PO_4_ (0 ppm), P0), with the available P contained in the mixed matrix 0.29 ± 0.02 mg·kg^−1^). Each P supply treatment was inoculated with *Glomus intraradices* (Gi) and no inoculation treatment (CK). Seven replicates were performed for each treatment; there were 28 pots in total.

The cultivation substrate was a mixture of river sand and perlite 3:1 (L/L). After washing and drying, the river sand was mixed with perlite by 2 mm mesh sieve. The cultivation substrate was put into a sterilization bag (high-density polyethylene film) with a diameter of 25 cm and a length of 40 cm and sterilized in a vertical-pressure steam sterilizer. The condition of each sterilization was 121°C (0.1 ~ 0.2 MPa); high-temperature sterilization was carried out for 30 min and then dried in a sterile environment for 7 days. Each pot was filled with 5.0 kg of the mixed substrate with fungal soil and cultivation substrate, following the volume ratio of 0.6:6 (L/L). The pH values of the cultivation substrate and the mixed substrate were 6.33 ± 0.12 and 6.59 ± 0.07, respectively, and the available P concentration was 0.21 ± 0.03 mg·kg^-1^ and 0.29 ± 0.02 mg·kg^-1^. The fungal soil containing the corresponding matrix, AM fungal spores, and extraradical hyphae were provided by the Institute of Plant Nutrition and Resources, Beijing Academy of Agricultural and Forestry Sciences. The spore density in this fungal soil was 30 spores/10g.

To satisfy the tested seedling requirements for other nutrients, each pot was supplied with a quarter of the modified Hoagland nutrient solution formula (Wu et al., 2011). Each time with 60 ml every 7 days. The potassium (K^+^) concentration of all treatments was adjusted to a similar level by adding KCl, and 200 ml of pure water was poured every 5 days in the afternoon. The temperature in the greenhouse was 18-28°C; the average photoperiod was 10 h day^−1,^ and relative humidity was >80%. The tested seedlings were harvested after 90d.

### 2.2 Data collection and statistical analysis

The root staining procedure was modified from the Trypan Blue staining method (Phillips and Hayman, 1970). The decolorized root segments were placed on slides, pressed with coverslips, and placed under a light microscope with a 10× optical lens for observation. The root infection rate of *G. intraradices* (Gi) (*F*/%) was calculated using “MYCOCALC” software with the following formula. As long as mycorrhizal structures such as hyphae, vesicles, and arbuscular appear in the root segments, the roots are considered to have been colonized by Gi (Xie et al., 2014).


F=(number of mycorrhizal infested segments/ number of all root segments)×100%

Under low P stress, plants tend to dissolve root cortical cells to form cavities, which were quickly occupied by air to become aerenchyma, which was conducive to reducing the respiratory consumption of old root tissues in stress environment; besides, the P nutrients and other compounds dissolved in the cortex could be transported to other parts, which was also beneficial to plant growth and development (Postma and Lynch, 2011). A large number of studies have shown that the formation of RCA could be induced, and previous studies have shown that RCA may play an important role in reducing the metabolic cost of P-deficient plants to obtain resources (Wu et al., 2018).

The sections of root segments prepared using the freehand sectioning method were observed under a microscope (10×) and then analyzed using Image J 1.46e software for root cross-sections, mid-column, and area of cortical dissolution to form aeration tissue. The root cortical tissue dissolution rate (%) was calculated as the dissolution area (μm^2^)/the total root cortical area (μm^2^).

The root length, surface area, root volume, and average root diameter of the root system were quantified and analyzed using the WinRHizo (version 4.0B) root analysis system software after harvesting the seedlings. After harvesting, the seedlings were harvested separately by root and aboveground parts, weighed, placed in an oven at 105°C for 30 min, and dried at 80°C until constant weight. The aboveground and root biomass were measured separately to calculate the root-to-shoot ratio.

The relative field mycorrhizal dependency (RFMD) index was used to descript the mycorrhizal dependency of the tested Chinese fir seedlings under two P-supplying treatments, which was calculated as follows (Plenchette et al., 1983),


$$\begin{array}{l} \text{RFMD}=\{(\text{dry mass of the mycorrhizal seedlings}-\text{dry mass of the non}-\text{mycorrhizal seedlings})\\ /\text{dry mass of the mycorrhizal seedling}\}\times 100\% \end{array}$$

The dried samples were crushed, sieved and treated, and then the samples were digested by the H_2_SO_4_-HClO_4_ decoction method, and the P content of each organ was determined by the molybdenum antimony anti-colorimetric method; the P use efficiency of each organ was expressed as the ratio of the biomass of each organ to the P accumulation of each organ.

Two-way ANOVA was used on the experimental data to analyze whether two factors have significant interaction performed using SPSS 25.0. Significant comparisons were made using an independent samples t-test, Duncan’s multiple comparison method (*p* = 0.05). All data were expressed as mean ± standard error (SE), and correlation charts were drawn using Origin 2021.

## 3 Results

### 3.1 Infection rate of Gi in the root system of Chinese fir seedlings

**Figure 1:**
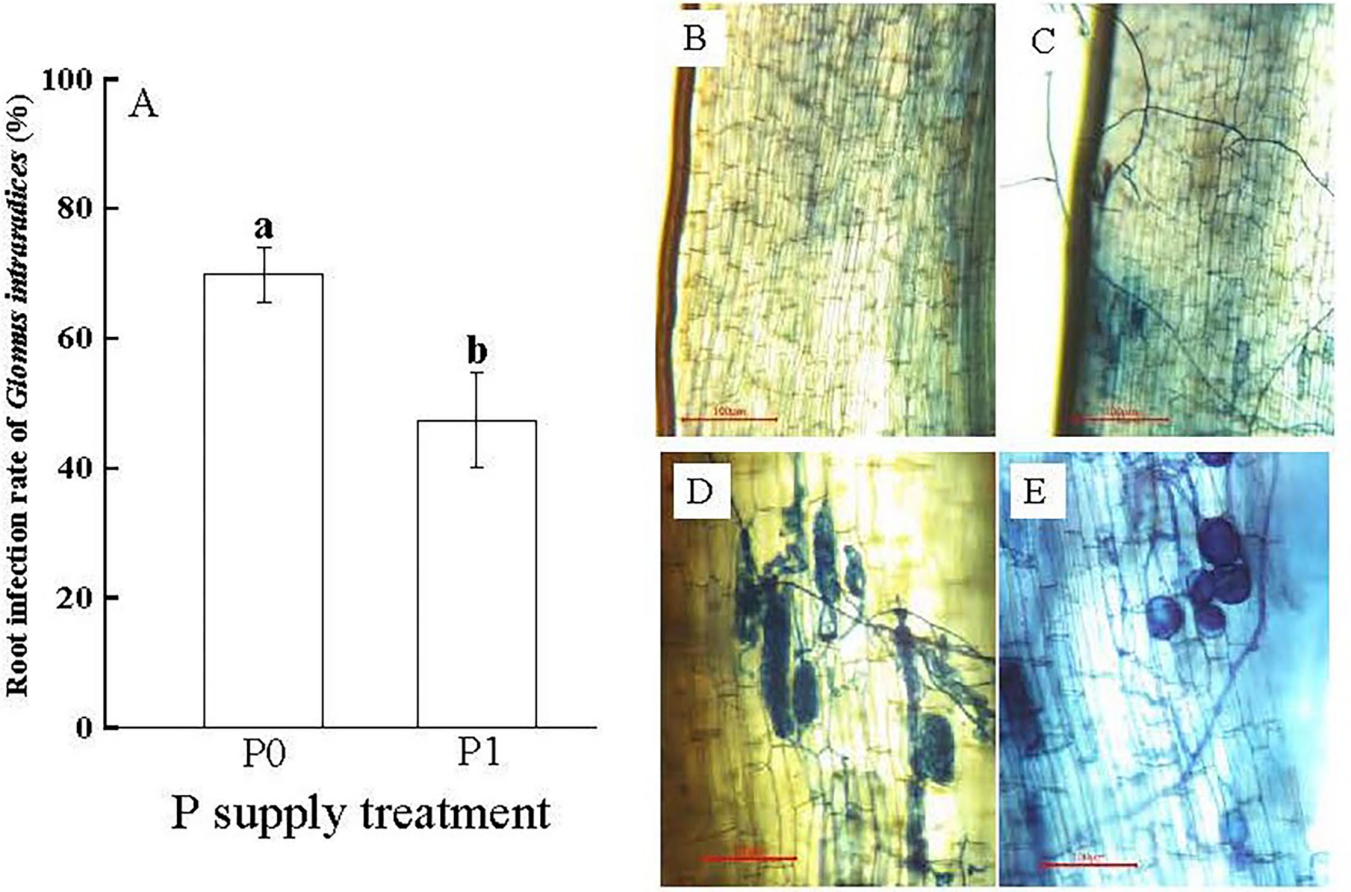
**(A)** The root infection rate of *Glomus intraradices* Gi of Chinese fir under different phosphorus treatments. In the figure, P0 represents the no phosphorus supply, P1 represents the normal phosphorus supply, and the different lowercase letters indicate significant differences between the two treatments (*p<* 0.05). **(B–E)** show the microscopic observations of arbuscular mycorrhizal fungi infecting the roots of Chinese fir seedlings, no Gi infection was found in **(B)**. Different processes of Gi infection in Chinese fir roots were found in **(C–E)**.

The treatment without P supplying (P0) significantly promoted Gi infestation in the Chinese fir seedling root systems (*p<* 0.05). Gi infection rate was (69.81 ± 4.29%) in the P0 treatment, which was 1.47 times higher than that in the P1 treatment (**Figure 1A**). The different proceeding of infestation of Gi was observed, including the mycelium (**Figure 1C**), the arbuscular morphological structure (**Figure 1D**), the vesicles and spore (**Figure 1E**), compared with non-infected root tissues (**Figure 1B**). Furthermore, compared to the P1 treatment, the RFMD in the P0 treatment was 6.12%, which was 2.72 times higher than P1 (**Table 1**).

**Table 1:** Dry weight and calculated mycorrhiza dependence of Chinese fir seedlings inoculated with Gi and without Gi under two phosphorus supply treatments, respectively.

P supply treatment	Dry weight of mycorrhizal seedlings (g)	Dry weight of non-mycorrhizal seedlings(g)	Relative field mycorrhizal dependency (RFMD) index (%)
P0	6.00	5.63	6.12
P1	5.86	5.73	2.25

Yields were evaluated by weighing the harvested seedling.

### 3.2 Root morphological plasticity

**Figure 2:**
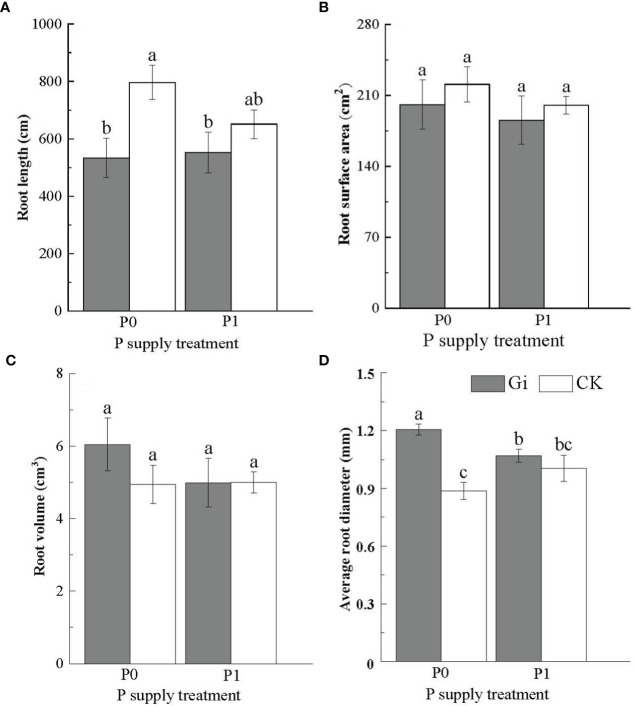
Effects of arbuscular mycorrhizal fungi inoculation with different phosphorus supply treatments on the root morphology of Chinese fir seedlings. In the figure, **(A–D)** represent the changes of root length, root surface area, root volume and average root diameter when inoculated with Gi under different phosphorus treatments. P0 represents the no phosphorus supply, and P1 represents the normal phosphorus supply. Gi represents the inoculation with Gi, and CK represents no inoculation of Gi. The different lowercase letters indicate significant differences between the two treatments (*p<* 0.05).

The root length of Chinese fir was significantly reduced (*p<* 0.05) after Gi inoculation compared to the non-inoculated treatment under the P0 treatment. However, no significant difference was observed in the root length between the Gi-inoculated and non-inoculated treatment under the P1 treatment (*p* > 0.05) (**Figure 2A**). Additionally, there was no significant difference (*p* > 0.05) observed between the root surface area (**Figure 2B**) and root volume (**Figure 2C**) of Chinese fir inoculated with Gi and the non-inoculated treatments under P0 and P1 treatments. The average root diameter reached 1.21 ± 0.03 mm after Gi inoculation under the P0 treatment, which was 1.35 times greater than the non-inoculated treatment (**Figure 2D**). Compared with the P1 treatment, the average root diameter of Chinese fir inoculated with Gi under the P0 treatment condition was significantly larger (*p<* 0.05). Two-way ANOVA results are shown in (**Table 2**).

**Table 2:** The results of two-factor (phosphorus supply treatment and inoculation treatment) variance analysis of phosphorus use efficiency, root morphology, and biomass-related parameters.

Trait	F value		
	P supply level (a)	AMF (b)	a×b
Plant P use efficiency	9.622^**^	0.899	0.006
Shoot P use efficiency	8.131^**^	0.611	< 0.001
Root P use efficiency	13.338^**^	4.061	0.029
Root length	1.034^**^	8.403^**^	1.722
Root surface area	0.851	0.785	0.019
Root volume	0.744	0.882	0.902
Average root diameter	0.032	17.358^**^	7.581^*^
Plant biomass	0.230	1.623	0.016
Shoot biomass	0.830	1.924	0.581
Root biomass	0.517	2.295	0.501
Root-to-shoot ratio	3.800	9.208^**^	0.293

* and ** represents significance level at (*p<* 0.05) and (*p<* 0.01) respectively.

### 3.3 Plant biomass production and allocation

**Figure 3:**
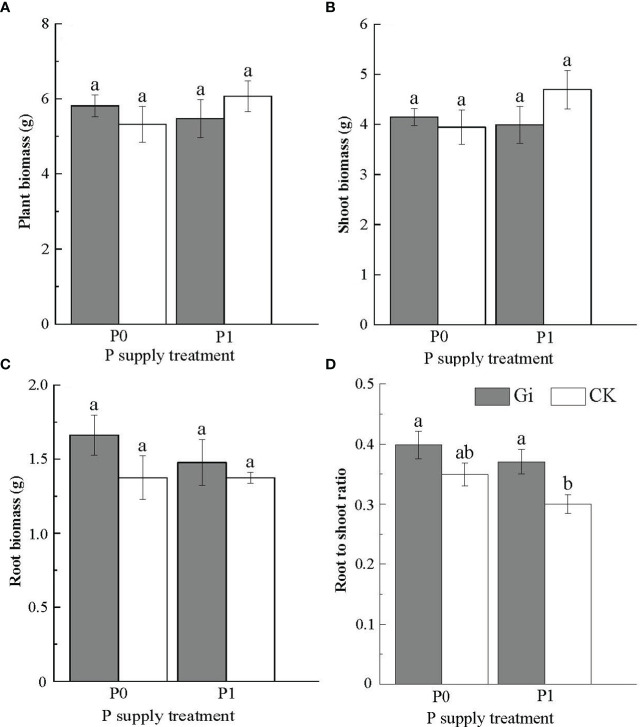
Effects of arbuscular mycorrhizal fungi inoculation with different phosphorus supply treatments on the biomass of Chinese fir seedlings. In the figure, **(A–D)** represent the changes of plant biomass, shoot biomass, root biomass and root to shoot ratio when inoculated with Gi under different phosphorus treatments. P0 represents the no phosphorus supply, and P1 represents the normal phosphorus supply. Gi represents the inoculation with Gi, and CK represents no inoculation of Gi. The different lowercase letters indicate significant differences between the two treatments (*p<* 0.05).

The differences in biomass of Chinese fir between treatments did not reach a significant level (*p* > 0.05, **Figures 3A–C**). The root-to-shoot ratio inoculated with Gi increased significantly (*p<* 0.05) under the P1 treatment compared to the non-inoculated treatment (**Figure 3D**).

### 3.4 Phosphorous use efficiency and cortical tissue dissolution

**Figure 4:**
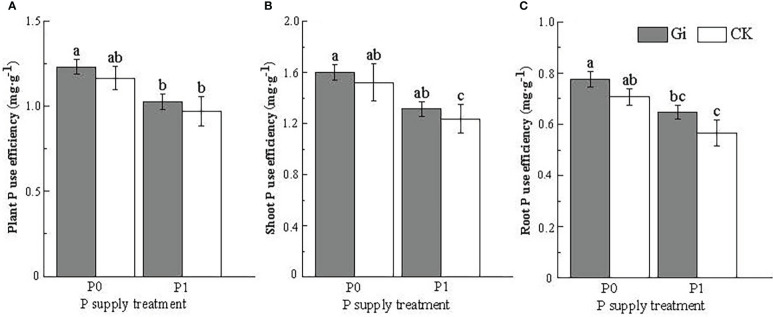
Effects of arbuscular mycorrhizal fungi inoculation with different phosphorus supply treatments on the P use efficiency of Chinese fir seedlings. In the figure, **(A–C)** represent the changes of plant P use efficiency, shoot P use efficiency, root P use efficiency, when inoculated with Gi under different phosphorus treatments. P0 represents the no phosphorus supply, and P1 represents the normal phosphorus supply. Gi represents the inoculation with Gi, and CK represents no inoculation of Gi. The different lowercase letters indicate significant differences between the two treatments (*p<* 0.05).

After inoculation with Gi under P0 treatment, both plant P use efficiency (**Figure 4A**) and root and shoot P use efficiency (**Figures 4B, C**) of Chinese fir showed a significant increase (*p<* 0.05) compared to Gi inoculation under P1 treatment. For no Gi inoculation, the root P use efficiency of Chinese fir was also significantly increased under the P0 treatment compared to the P1 treatment (*p<* 0.05) (**Figure 4**).

**Figure 5:**
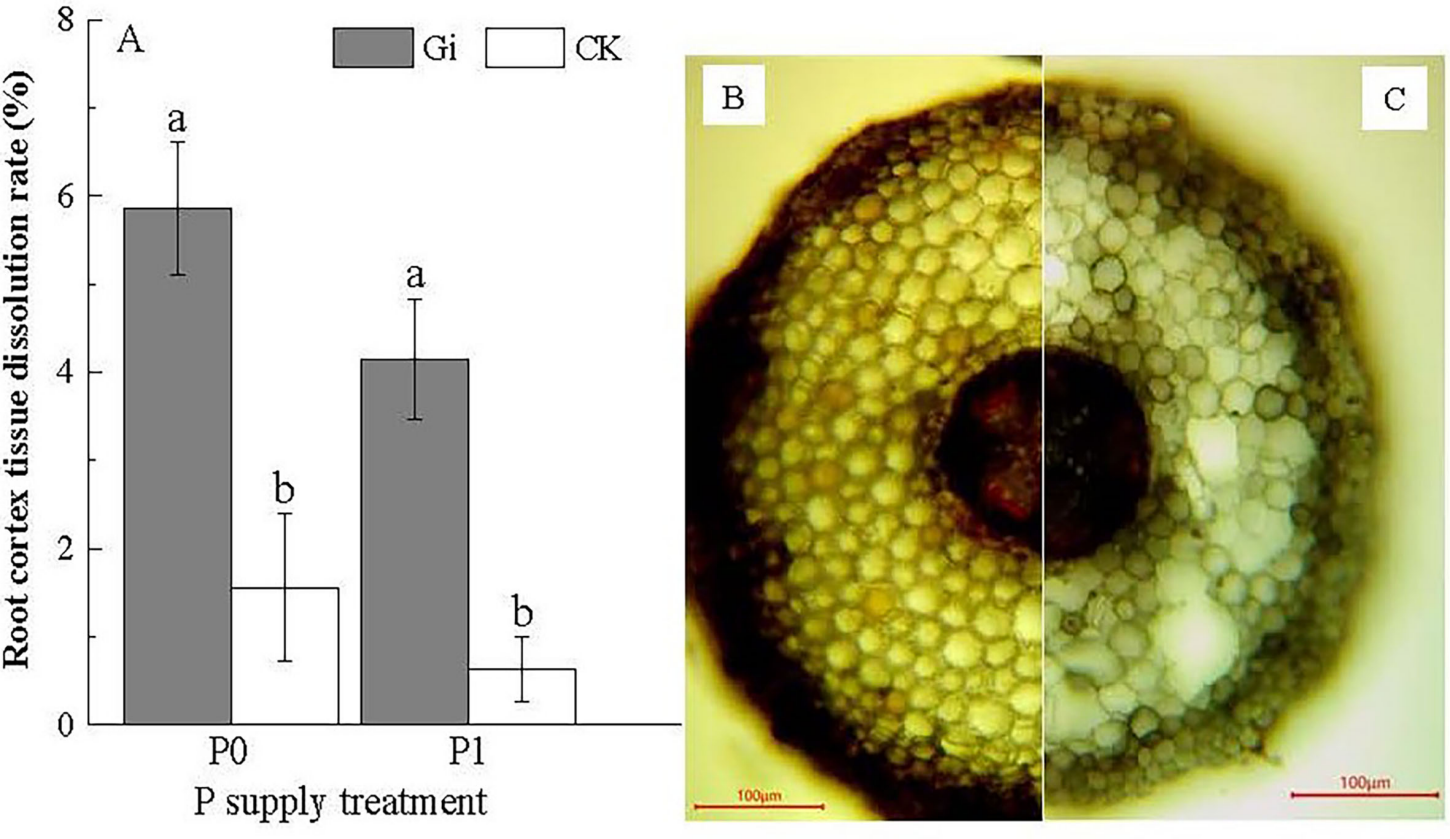
**(A)** The effect of *Glomus intraradices* Gi inoculation on the dissolution of root cortex tissue of Chinese fir under different phosphorus treatments. In the figure, P0 represents the no phosphorus supply, and P1 represents the normal phosphorus supply. Gi represents the inoculation with Gi, and CK represents the no inoculation of Gi. The different lowercase letters indicate significant differences between the two treatments (*p<* 0.05). **(B, C)** show the microscopic observation of Gi infecting the roots of Chinese fir seedlings. Compared with **(C)**, the cortex tissue of roots in **(B)** did not dissolve clearly.

Combined with the observation of Gi infestation on the root system of Chinese fir (**Figures 1C–E**), the root cortical tissue cells and mycelial structure of the root system after Gi infestation could be distinguished. According to **Figure 5**, the root cortical tissue dissolution rate reached upto 5.85 ± 0.76% after inoculation with Gi under P0 treatment, which was 3.75 times higher than CK treatment. Under the P1 treatment, the root cortical tissue dissolution rate after inoculation with Gi was (4.15 ± 0.68%), which was 6.59 times higher than that of the non-inoculated treatment. The treatment with Gi inoculation significantly promoted root cortical tissue dissolution under both P0 and P1 treatments (*p<* 0.05). Through microscopic observation, it can be seen that some Chinese fir cells have been dissolved in the root cortex (**Figure 5C**.)

## 4 Discussion

Plants have evolved through various strategies to resist environmental stresses during long-term natural selection (Lamalakshmi et al., 2017). Previous studies have shown that rhizosphere P concentration is one of the main factors affecting AMF infection in plants (Wang et al., 2010). The increase of soluble P concentration in the soil will reduce the fungal colonization of roots (Bi et al., 2003; Leung et al., 2010). In this study, the root infection rate of Gi under the no P supply treatment (P0) was significantly higher than that of the normal P supply treatment (P1). This action benefited trees to establish a mutually beneficial symbiosis with AMF to mitigate the effects of low P stress on its growth (Ramaekers et al., 2010). Numerous studies have shown that plant roots can resist low P stress through morphological and structural responses (Liang et al., 2014; Wu et al., 2017). In this study, the effects of P supply treatment and AMF inoculation on morphological indicators such as root length and average diameter of the root system of Chinese fir were more significant. Under the P0 treatment, the root length was significantly lower. The average root diameter was considerably higher after inoculation with AMF (Gi) than without Gi treatment, probably because the Chinese fir root system formed a mycorrhizal symbiosis with AMF, which reduced the input pathway of P foraging through its own fine root proliferation. The plant increased the material distribution to the root system for its material exchange with AMF and was supplied with P by the extensive mycelial network of AMF (Smith et al., 2000; Ferrol et al., 2019).

It is well known that P promotes root growth and vitality, and N, as the main component of protein, plays a vital role in the development of stems and leaves. Studies have shown that when nutrient availability is low, mangrove seedlings invest more resources to increase root biomass (Naidoo, 2009). Due to the large absorption of P by mycorrhiza, the ratio of N to P decreased, which may affect the growth of aboveground parts (Xie et al., 2014). On the other hand, plants try to balance the P uptake and consumption to maintain normal growth in a low P environment. The plant could adjust the allocation between the aboveground and root systems to reduce the metabolic cost of P foraging for the root system (Ramaekers et al., 2010; Haling et al., 2016). Under both P supply treatment conditions in this study, inoculation with AMF increased the root biomass to some extent. Among them, AMF inoculation significantly increased the root-to-shoot ratio of Chinese fir under P1 treatment conditions (*p<* 0.05). AMF inoculation under P0 treatment also increased the root-to-shoot ratio of Chinese fir, but this effect did not reach a significant level (*p* > 0.05). It may be that under low P stress, the plant root system can enhance its ability to form symbiotic structures with soil AMF. The plant can obtain P nutrient from AMF to support growth needs when providing AMF with C mass (Li et al., 2019; Ferrol et al., 2019).

In addition, the results showed that there was no significant difference in biomass between different treatments, which may be due to the short period of the pot experiment and the high physiological activity of seedlings (**Table 2**; **Figure S2**). Previous studies have shown that the clone seedlings can resist low P stress by adjusting root growth and secretion, and cortical tissue dissolution to maintain a suitable level of growth (Zou et al., 2018). The biomass changes of seedlings inoculated with Gi under different P treatments were analyzed. It was found that the biomass of seedlings inoculated with Gi increased compared with the non-inoculated Gi group under the P0 treatment; however, only the root biomass of seedlings inoculated with Gi increased compared with the non-inoculated Gi group under the P1 treatment. Combined with the analysis of the RFMD of seedlings under different phosphorus treatments, the results showed that the RFMD of Chinese fir seedlings were 6.12%^P0^ and 2.25%^P1^ under different P supply treatments, respectively. The biomass of seedlings at low P concentration depended more on mycorrhizal than the high P concentration.

Plants can maintain growth and development under low P conditions by increasing the P use efficiency in their bodies (Veneklaas et al., 2012; He et al., 2013), which is an important basis for measuring the ability of plants to cope with low P stress (Wu et al., 2011). In terms of the effect of P supply treatment and AMF inoculation on the P use efficiency of Chinese fir, under the condition of no AMF inoculation, the root P use efficiency of Chinese fir was significantly greater under P0 treatment compared to P1 treatment (*p<* 0.05, **Figure 4C**). Similar to this pattern, the root P use efficiency and whole plant P use efficiency of Chinese fir roots under no P supply stress increased significantly (*p<* 0.05, **Figures 4A**, **C**) after AMF inoculation of Chinese fir roots. High P use efficiency may be achieved by improving the reuse of P in roots and acquiring P by hyphae. It has been reported that plants in low P environments could also form a large amount of aeration tissue through cortical tissue dissolution to reduce root respiration and satisfy P demand to a certain extent (Wu et al., 2018). Under low P stress, cell lysis in the cortical tissue of the mature zone of Chinese fir roots was evident, and the released P nutrient was involved in recycling *in vivo* (Postma & Lynch, 2011). At the same time, cortical tissue cells lysed to form a larger space, facilitating AMF colonization and enhancing its ability to resist stress. In this study, we found that inoculation of AMF significantly promoted root cortical tissue dissolution under both P-supply treatment conditions. Among them, the root cortical tissue dissolution rate was the largest after inoculation with AMF under the P0 treatment, which was 3.75 times higher than that of the non-inoculated treatment. It indicates that when AMF colonizes Chinese fir roots, AMF transfers P captured by its mycelium to Chinese fir roots as Chinese fir provides living space and nutrients for it. This increases the P uptake of Chinese fir to some extent (Smith et al., 2004; Rooney et al., 2011) and improves its *in vivo* P use efficiency to be better used to maintain material exchange between mycorrhizal symbioses.

## 5 Conclusions

No P-supplying (P0) treatment considerably enhanced the Gi infection rate in the root system of Chinese fir seedlings. The radial root diameter growth was significantly promoted under low P stress, but root length extension ability was inhibited considerably after Gi inoculation. Inoculation of Gi under different P supply treatments promoted the growth and development of Chinese fir to some extent. No P supply treatment significantly increased root cortical tissue dissolution rate compared to normal P supply. Chinese fir may prefer to form a mutually beneficial symbiosis with AMF to obtain more of the P nutrient required for efficient growth, especially in low P environments. The exchange of organic matter and nutrients through mycorrhizae met the needs of AMF’s growth and enabled the Chinese fir to obtain P nutrients necessary for its organic matter synthesis from the mycorrhizal network.

## Data availability statement

The original contributions presented in the study are included in the article/**Supplementary Material**. Further inquiries can be directed to the corresponding author.

## Author contributions

PW, JX and YT conceived the idea and designed the experiment. YT, JX, XL, and BW conducted the study. YT and JX wrote the manuscript. XM give suggestions to the manuscript. PW provide the funding, supervise the experiment, reviewed the manuscript and contributed to the discussion. All authors contributed to the article and approved the submitted version.
