# Supplementary material for: Effect of Glomus intraradices on root morphology, biomass production and phosphorous use efficiency of Chinese fir seedlings under low phosphorus stress
Source: Front Plant Sci. 2023 Jan 6;13:1095772. doi: 10.3389/fpls.2022.1095772 (PMC9853428; doi:10.3389/fpls.2022.1095772)
Supplement: Supplementary file 1 [file DataSheet_1.docx]

# Supplement material

**Figure S1.** AMF colonization examined in the Chinese fir seedling roots before AMF inoculation treatment (no AMF colonization was examined)

**Figure S2.** Image scanning of seedling harvest after different treatments. It represents the harvest image of seedlings inoculated (Fig. S2-A) with Gi under P0 supply treatment; (Fig. S2-B) without Gi inoculation under P0 supply treatment; (Fig. S2-C) inoculated with Gi under P1 supply treatment; (Fig. S2-D) without Gi inoculation under P0 supply treatment

**Figure S1.**

**
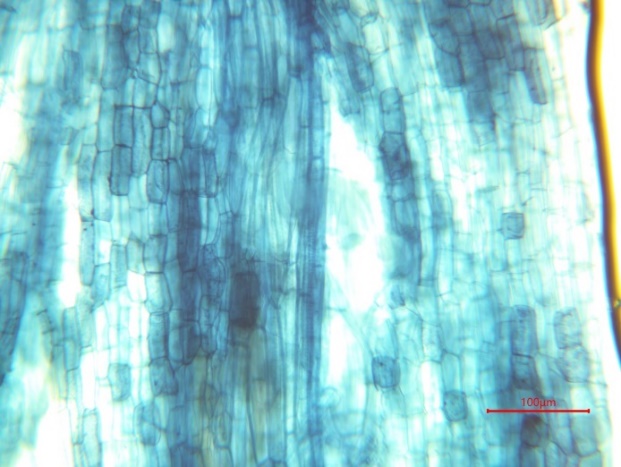
**

**Figure S2.**

**
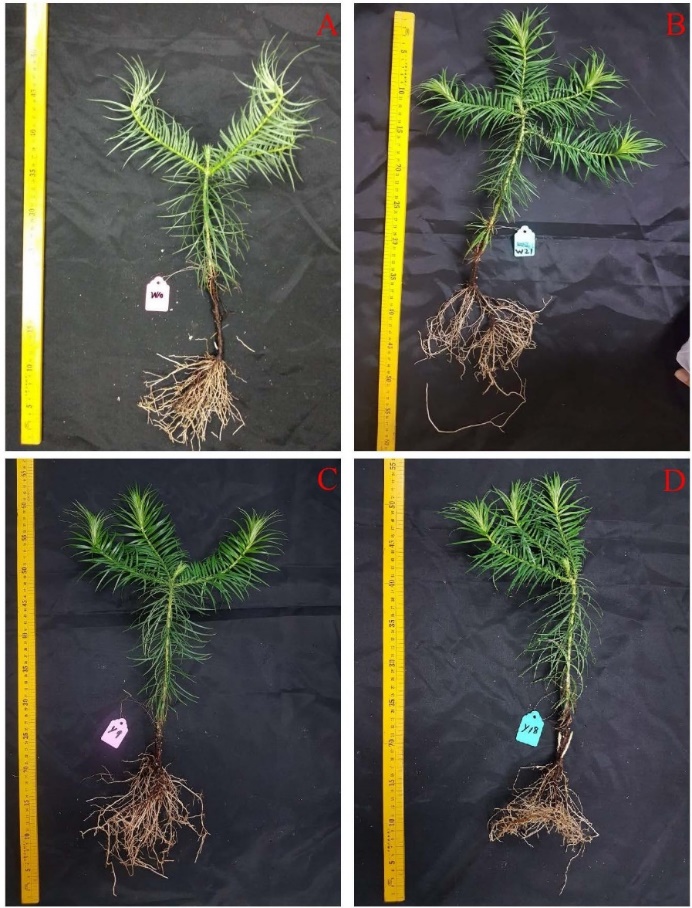
**
